# Differential effects of wake promoting drug modafinil in aversive learning paradigms

**DOI:** 10.3389/fnbeh.2015.00220

**Published:** 2015-08-19

**Authors:** Bharanidharan Shanmugasundaram, Volker Korz, Markus Fendt, Katharina Braun, Gert Lubec

**Affiliations:** ^1^Department of Pharmaceutical Chemistry, University of ViennaVienna, Austria; ^2^Institute for Pharmacology and Toxicology, and Center for Behavioral Brain Sciences, Otto-von-Guericke University MagdeburgMagdeburg, Germany; ^3^Department of Zoology/Developmental Neurobiology, Institute of Biology, Otto-von-Guericke University MagdeburgMagdeburg, Germany

**Keywords:** feedback learning, cognitive enhancer, two-way active avoidance, fear conditioning, aversive learning

## Abstract

Modafinil (MO) an inhibitor of the dopamine transporter was initially approved to treat narcolepsy, a sleep related disorder in humans. One interesting “side-effect” of this drug, which emerged from preclinical and clinical studies, is the facilitation of cognitive performance. So far, this was primarily shown in appetitive learning paradigms, but it is yet unclear whether MO exerts a more general cognitive enhancement effect. Thus, the aim of the present study in rats was to extend these findings by testing the effects of MO in two aversive paradigms, Pavlovian fear conditioning (FC) and the operant two-way active avoidance (TWA) learning paradigms. We discovered a differential, task-dependent effect of MO. In the FC paradigm MO treated rats showed a dose-dependent enhancement of fear memory compared to vehicle treated rats, indicated by increased context-related freezing. Cue related fear memory remained unaffected. In the TWA paradigm MO induced a significant decrease of avoidance responses compared to vehicle treated animals, while the number of escape reactions during the acquisition of the TWA task remained unaffected. These findings expand the knowledge in the regulation of cognitive abilities and may contribute to the understanding of the contraindicative effects of MO in anxiety related mental disorders.

## Introduction

Modafinil (MO; 2-[(diphenylmethyl) sulfinyl] acetamide), an inhibitor of the dopamine transporter, is a wake-promoting drug, which was approved by the Food and Drug Administration (FDA) in 1998 for treating excessive daytime sleepiness in narcolepsy and other sleep disorders, such as shift work sleep disorder and obstructive sleep apnea syndrome (Minzenberg and Carter, 2008). Further, the symptoms of certain psychiatric disorders such as major depressive disorder (Kaufman et al., 2002), schizophrenia (Turner et al., 2004), Parkinson’s disease (PD; Nieves and Lang, 2002), and attention deficit hyperactivity disorder (Taylor and Russo, 2000) can be relieved by MO. The underlying mechanisms of the pro-cognitive effects of MO that are paralleled by enhanced alertness, attention, and accuracy (Minzenberg and Carter, 2008) are still widely unknown. A large body of studies using rodents in different memory tasks and behavioral states such as spatial working and long-term memory, attention, impulsive behavior, speed of response and accuracy, support the evidence of cognitive enhancing effects (Minzenberg and Carter, 2008).

So far, the cognitive enhancing effects of MO were mainly analyzed in appetitive learning paradigms, based on the assumption that the release of dopamine is a key modulator of reward related learning. Chronic administration of a 75 mg/kg MO dose prior to training in mice and a 10 and 64 mg/kg dosage in rats dose-dependently improved the acquisition in a Morris water maze task (Shuman et al., 2009; Tsanov et al., 2010) and in a multiple T-maze task. (Sase et al., 2012). In a spatial serial discrimination task using a holeboard apparatus MO at 16 and 32 mg/kg doses prior to the test phase had no effect whereas in contextual serial discrimination task, 32 but not 16 mg/kg dose enhanced performance in both, healthy and sleep deprived mice (Béracochéa et al., 2008; Pierard et al., 2011). A much higher dose of 75 mg/kg in an object recognition task in naive rats however had no effects on memory consolidation or retrieval (its effect on acquisition was not tested; Garcia et al., 2013), whereas 55 and 100 mg/kg dosages resulted in significantly more correct choices in delayed nonmatching to position task in rats (Ward et al., 2004). This emphasizes that the kind of task, the rodent species, the dosage and the duration of application is critical.

In contrast, only a few studies employed aversive avoidance learning paradigms to investigate whether MO exerts similar effects as revealed for reward-based learning tasks. Memory consolidation or retrieval of a *passive* avoidance task using the step-down avoidance test in rats, remained unaffected by MO when administered immediately after training or 1 h before testing on day-2, while possible effects of MO on acquisition were not tested (Garcia et al., 2013). MO effects in another *passive* learning paradigm, fear conditioning (FC), revealed MO modulated immediate freezing response during acquisition as well as context related freezing (Shuman et al., 2009). Experiments in a plus-maze discriminative avoidance task conducted with mice revealed that MO exerts a highly dose-dependent aggravation in learning: a high dosage of MO induced memory impairments in mice, 32 mg/kg administered before training impaired memory retrieval, whereas 64 and 128 mg/kg blocked memory consolidation (Fernandes et al., 2013).

Throughout the literature very high doses of MO (32–200 mg/kg) were used in rodents for testing its action in various memory paradigms (Minzenberg and Carter, 2008). These doses are not clinically relevant and the reported diverse and contradictory findings may be induced by severe side effects like increased arousal, locomotor activity etc. Psychostimulants like methylphenidate, amphetamines were known to enhance cognition at low doses with no apparent side effects (Wood et al., 2014).

Thus, due to the paucity of studies and high concentration of MO used in these studies, and in view of the differential effects described for MO in the different learning paradigms, the aim of this study was to explore the effects of MO in two aversive learning paradigms, in a simple Pavlovian FC paradigm and in a more complex two-way avoidance (TWA) operant conditioning paradigm using clinically relevant low doses. So far, MO effects have not been tested in TWA *active* avoidance paradigm.

Pavlovian FC is a widely studied memory paradigm in which an aversive unconditioned stimulus (UCS), usually a foot shock, is paired with an initially neutral conditioned stimulus (CS), a tone or light pulse. The UCS normally provokes an unconditioned defensive reaction. Following temporal coupling of the UCS and the CS through training, the CS alone elicits a conditioned fear response. Apart from making an association between UCS and CS as a tone (cue association), the animal can also associate the context i.e., the experimental environment (test apparatus), odor etc., which serve as additional CS (context association). Learning and memory retrieval is measured and quantified by measuring the duration of freezing, i.e., the lack of any movement apart from breathing, and the duration of freezing is considered proportional to the strength of association (Curzon et al., 2009).

In humans and other animals positive as well as negative feedback is essential to optimize behavioral strategies. TWA is a type of feedback-based learning, which requires the ability to incorporate performance feedback into the learning process. The TWA task is a negative reinforcement instrumental conditioning paradigm analyzed in young and adult mice (Spröwitz et al., 2013) and rats (Schäble et al., 2007; Gruss et al., 2010; Riedel et al., 2010) in which the rodent has to learn a complex strategy to avoid electric shocks, widely used to test psychoactive drugs during drug screening (Goswami et al., 1996; Getova et al., 2003). Microdialysis experiments from medial prefrontal cortex of gerbils during TWA demonstrated a transient increase of dopamine efflux correlated with the establishment of avoidance behavior (Stark et al., 2004).

## Materials and Methods

### Animal Housing

Female Wistar rats were used for this study. For the TWA test we used eighty rats in total: 20 rats in each for the 10 mg/kg dose group and the related vehicle group. 10 rats in each for the 5 and 1 mg/kg dose groups and its related vehicle groups. For the FC experiment, we used 12 rats in each for the vehicle and the drug treated groups. Animals were purchased from Janvier labs, France and housed in groups of five in translucent standard laboratory cages type IV (E. Becker and Co. GmbH, Castrop-Rauxel, Germany) and reared under normal animal facility conditions (temperature: 22 ± 2°C; humidity: 55 ± 5%; 12 h artificial light/12 h dark cycle: light on at 6:00 am) with *ad libitum* access to food (Altromin 1320; Lage, Germany) and water. We prefer to work with females here because in TWA paradigms, our previous work has demonstrated that females are on average better learners and show smaller individual variability (Schäble et al., 2007; Gruss et al., 2010) and moreover there is no influence of the estrous cycle (Rubio et al., 1999). The animals were allowed to accommodate in the institute’s animal house for at least 2 weeks prior to the onset of the experiments, when the animals were 11–14 weeks old. All experiments were performed in accordance with international ethical guidelines for the care and use of laboratory animals in experiments (2010/63/EU) and were approved by the local authorities (Landesverwaltungsamt Sachsen-Anhalt). Prior to the start of the experiments the rats were handled for 5 min/day for 3 days to habituate to the experimental procedures. All behavioral experiments were performed between 8:00 am and 2:00 pm.

### Drug Administration

MO synthesized in our laboratory was freshly dissolved in volumes as small as possible in 100% dimethyl sulfoxide (DMSO), since MO does not dissolve in aqueous solvent. The rats received intraperitoneal injections of vehicle or 500 μl/kg drug at doses of 1, 5 and 10 mg/kg. In humans, typically 100–400 mg MO doses are used to treat various sleep disorders (about 1–3 mg/kg) which produces a plasma concentrations of 1–4 μg/ml (Ballon and Feifel, 2006; Guo et al., 2010). We did pharmacokinetic studies of MO in rats and found 10 mg/kg i.p. injection of MO produced 1 μg/ml MO in blood plasma 30–60 min after administration (data not shown). Thus, we set the maximum dose to 10 mg/kg and even two lower doses 5 mg/kg and 1 mg/kg were used for our studies. Since the drug was administered in low doses, we used a non-oral route which is generally considered to be more effective with high bioavailability of the drug. The vehicle/drug was injected daily for 5 days 30 min prior to the TWA test. In the FC test vehicle and drug was injected 30 min before the start of the acquisition only at day-1. We observed sometimes in a few cases a writhing response and vocalization immediately after the injection, which lasted up to 10 s.

### Fear Conditioning Setup

FC was conducted in the same apparatus as used for TWA but confined to one compartment with the connecting door closed. A video camera was mounted to the wall facing the conditioning chamber. The chambers were cleaned with 70% ethanol between trials. The protocol used in this study was adopted from Wood and Anagnostaras (2011) with minor changes.

Training (day-1) consisted of a 2 min baseline period (habituation), followed by two tone shock pairing with an inter-trial interval (ITI) of 20 s. The tone was for 15 s (2.4 kHz, 85 dBA), co-terminating with a scrambled, constant current AC footstock (2 s, 0.6 mA, RMS). Immediate post-shock freezing during post shock period (PSP) was measured for 3 min, resulting in around 6 min total exposure to the training context. To measure *contextual fear learning* the rats were returned to the conditioning context on day-5 and freezing was recorded for 5 min with no tone or shock presented.

**Figure 1:**
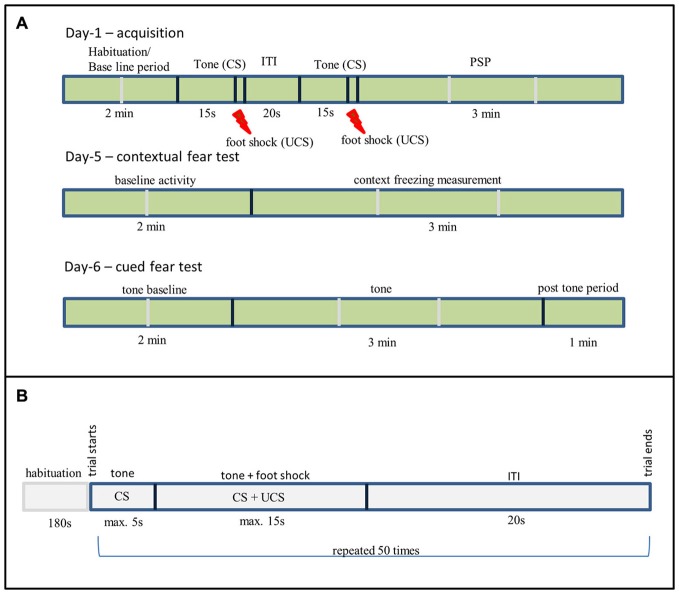
**(A)** Fear conditioning (FC) experimental setup, ITI—Inter-Trial Interval, PSP—Post Shock Period, Shock—0.6 mA for 2 s. **(B)** Two way active avoidance experimental setup.

To assess *cued conditioning* (tone) an altered context was used that differed in dimension (450 × 270 × 230 mm^3^), color (solid blue on all sides except front), flooring (white rugged), and odor (5% white vinegar solution) from the box that was used for the context conditioning. On one side of the box the wall was transparent that allows video camera to record the experiment. The rats were placed in the non-conditioning context on day-6 (24 h after context test), the testing consisted of a 2 min baseline period (baseline activity), followed by a 3 min tone identical to the tone used in training and followed by additional 1 min post tone monitoring. No shock was delivered during the cued freezing testing phase. Freezing was used as the dependent measure for both tests. The relatively long gap between training and re-testing in the FC paradigm was chosen to allow for drug clearance and the resumption of a normal sleep-wake cycle. The experimental setup is shown in the Figure 1A.

### Two-Way Active Avoidance Paradigm

The training and testing was conducted in a shuttle box (TSE Systems, Germany) as described in previous publications (Riedel et al., 2010). Rats were trained for five consecutive days; each training day consisted of 50 trials. The following parameters were set for the each trial. The CS is a 2.4 kHz tone given for 5 s, followed by a simultaneous application of UCS, which was a 0.6 mA foot shock applied for a maximum duration of 15 s after which the tone and the shock co-terminates. The trials were separated by a 20 s ITI. The training day started with a 3 min habituation period during which the rats freely explores the box. The experimental settings are shown in Figure 1B. The following behavioral responses shown by animals were recorded. Learning success was measured by assessing the number of *avoidance reactions*: the rat moves into the other compartment after the onset of CS but prior to UCS. *Escape reaction*: the rat changes the compartment after the UCS onset. If the rats do not change the compartment before the termination of CS and UCS it was counted as *failures*. The following parameters were recorded for each training day: number of avoidances, escapes and failures, and avoidance and escape latencies for each rat. The shuttle box was cleaned with 70% ethanol (Roth, Germany) after completion of all trials to remove odor cues.

### Statistical Analysis

The statistical analysis was performed using SPSS (version 20.0; SPSS Inc., Chicago, IL, USA). For comparing the drug effects on freezing during ITI and PSP of acquisition phase, contextual freezing and cued freezing independent sample *t*-test (for parametric data) or Mann-Whitney *U* test (for non-parametric data) was used. For comparing the drug effects on different TWA parameters (avoidances, escapes, failures, avoidance latency, escape latency), general linear model for repeated measure one way analysis of variance (ANOVA) was applied with drug treatment as main factor and training days (day 1–5) as repeated measure. For a detailed day-by-day analysis of drug treatment effects on TWA parameters, independent sample *t*-test was carried out. All tests were two-tailed and the significance level was set to *p* < 0.05. All data were presented as mean ± SEM in graphs.

**Table 1:** **The statistical output, *F* ratios and *p* values obtained from the analysis of TWA parameters using one-way repeated measures ANOVA**.

		Avoidances	Escapes	Failures	Avoidance latency	Escape latency
Effect of training	10 mg/kg	*F*_(2.79,108.80)_ = 44.75,	*F*_(2.77,108.06)_ = 43.17,	*F*_(2.69,105.13)_ = 3.26,	*F*_(2.88,95.22)_ = 4.22,	*F*_(2.9,114.51)_ = 23.31,
		*p* < 0.001	*p* < 0.001	*p* < 0.05	*p* < 0.005	*p* < 0.001
	5 mg/kg	*F*_(2.83,51.02)_ = 21.54,	*F*_(2.96,53.39)_ = 23.95,	*F*_(2.58,46.55)_ = 1.05,	*F*_(4,72)_ = 6.32,	*F*_(2.27,40.88)_ = 5.09,
		*p* < 0.001	*p* < 0.001	*p* = 0.372	*p* < 0.001	*p* < 0.005
	1 mg/kg	*F*_(4,72)_ = 47.58,	*F*_(2.58,46.56)_ = 14.49,	*F*_(2.21,39.88)_ = 5.65,	*F*_(2.18,39.26)_ = 2.24,	*F*_(2.27,40.91)_ = 10.76,
		*p* < 0.001	*p* < 0.001	*p* < 0.01	*p* = 0.115	*p* < 0.001
Interaction effect of drug treatment and training	10 mg/kg	*F*_(2.79,108.80)_ = 0.37,	*F*_(2.77,108.06)_ = 0.44,	*F*_(2.69,105.13)_ = 1.13,	*F*_(2.88,95.22)_ = 0.10,	*F*_(2.9,114.51)_ = 0.20,
		*p* = 0.758	*p* = 0.707	*p* = 0.337	*p* = 0.955	*p* < 0.8
	5 mg/kg	*F*_(2.83,51.02)_ = 0.68,	*F*_(2.96,53.39)_ = 1.28,	*F*_(2.58,46.55)_ = 0.57,	*F*_(4,72)_ = 1.26,	*F*_(2.27,40.88)_ = 0.70,
		*p* = 0.558	*p* = 0.284	*p* = 0.609	*p* = 0.293	*p* = 0.523
	1 mg/kg	*F*_(4,72)_ = 1.54,	*F*_(2.58,46.56)_ = 0.49,	*F*_(2.21,39.88)_ = 1.06,	*F*_(2.18,39.26)_ = 0.22,	*F*_(2.27,40.91)_ = 1.66,
		*p* = 0.199	*p* = 0.660	*p* = 0.361	*p* = 0.816	*p* = 0.199
Comparison between vehicle and MO groups	10 mg/kg	*F*_(1,39)_ = 12.85,	*F*_(1,39)_ = 10.50,	*F*_(1,39)_ = 8.75,	*F*_(1,33)_ = 0.12,	*F*_(1,39)_ = 4.60,
		*p* = 0.345	*p* = 0.296	*p* = 0.894	*p* = 0.797	*p* = 0.429
	5 mg/kg	*F*_(1,18)_ = 0.93,	*F*_(1,18)_ = 1.15,	*F*_(1,18)_ = 0.02,	*F*_(1,18)_ = 0.07,	*F*_(1,18)_ = 0.66,
		*p* = 0.345	*p* = 0.296	*p* = 0.894	*p* = 0.797	*p* = 0.429
	1 mg/kg	*F*_(1,18)_ = 0.48,	*F*_(1,18)_ = 1.95,	*F*_(1,18)_ = 0.99,	*F*_(1,18)_ = 0.38,	*F*_(1,18)_ = 0.96,
		*p* = 0.497	*p* = 0.179	*p* = 0.334	*p* = 0.546	*p* = 0.340

## Results

### Modafinil Dose Dependently Enhances Long-Term Memory for Contextual but not for Cued Fear Conditioning

**Figure 2:**
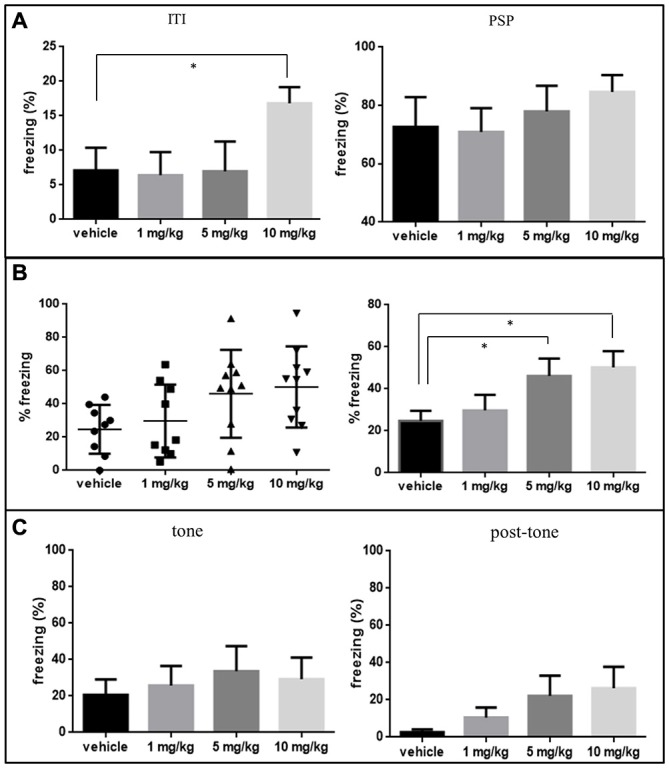
**(A)** The mean of freezing time expressed in percentage of the total time ± SEM of 1, 5 and 10 mg/kg MO treated groups in inter-trial interval (ITI) and in post shock period (PSP—last 2 min) on day-1 conditioning phase are shown in the bar graph. 10 mg/kg group was statistically significant in ITI, compared using two-tailed unpaired *t*-test. *p < 0.05 (vehicle vs. MO). **(B)** Freezing behavior of MO and vehicle treated rats during the “context freezing measurement” phase of contextual fear test, percent freezing time (mean ± SEM) expressed in distribution plot in the upper panel and bar graph in the lower panel. MO treated groups at dosages of 5 and 10 mg/kg showed more freezing behavior significantly compared to vehicle controls (Mann Whitney *U* test, *p < 0.05). **(C)** The mean of freezing percentage ± SEM of 1, 5 and 10 mg/kg MO treated groups in “tone” phase and “post-tone” phase of the cued freezing test.

During FC on day-1, rats treated with 10 mg/kg MO showed significantly higher freezing responses during the ITI (*p* < 0.05), but not during the PSP as compared to vehicle controls shown in Figure 2A. No freezing was observed during the base-line period in any of the groups (data not shown).

During contextual conditioning test, freezing behavior was significantly increased in animals which received 5 and 10 mg/kg compared to vehicle treated rats (*p* < 0.05) and there was no significant change in the 1 mg/kg MO treated groups (Figure 2B). For cued FC no significant effect for MO was observed during “tone” or “post tone” period for any of the groups (Figure 2C).

### Modafinil Dose Dependently Impairs Avoidance Learning in the Two-Way Active Avoidance Task

The analysis of avoidances for drug and vehicle treated groups during training revealed a significant increase of the number of avoidance responses with the respect to training days for all groups 1 mg/kg (*F*_(4,72)_ = 47.58, *p* < 0.001), 5 mg/kg (*F*_(2.83,51.02)_ = 21.54, *p* < 0.001) and 10 mg/kg (*F*_(2.79,108.80)_ = 44.75, *p* < 0.001) indicating a learning and memory effect. This is also indicated by a decrease of escape latencies, which significantly decreased during training in the 1 mg/kg (*F*_(2.9,114.51)_ = 23.31, *p* < 0.001), 5 mg/kg (*F*_(2.27,40.88)_ = 5.09, *p* < 0.005) and the 10 mg/kg (*F*_(2.27,40.91)_ = 10.76, *p* < 0.001) groups and also the number of failures decreased in 1 mg/kg (*F*_(2.21,39.88)_ = 5.65, *p* < 0.01) and 5 mg/kg (*F*_(2.69,105.13)_ = 3.26, *p* < 0.05) during training. The number of escape reactions significantly decreased in all groups 10 mg/kg (*F*_(2.77,108.06)_ = 43.17, *p* < 0.001), 5 mg/kg (*F*_(2.96,53.39)_ = 23.95, *p* < 0.001) and 1 mg/kg (*F*_(2.58,46.56)_ = 14.49, *p* < 0.001). However, avoidance latency significantly increased in 5 mg/kg (*F*_(4,72)_ = 6.32, *p* < 0.001) and 10 mg/kg (*F*_(2.88,95.22)_ = 4.22, *p* < 0.005) groups.

There was no significant drug treatment × training interactions effect in the any of the TWA parameters, indicating a similar shape of learning curves in drug treated as in vehicle treated groups (Table 1).

**Figure 3:**
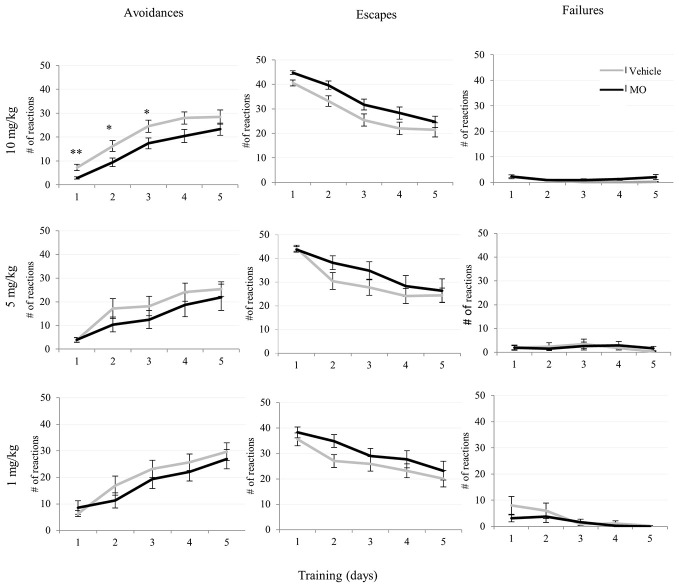
**Effects of MO at 10, 5, 1 mg/kg dosages on avoidance, escape and failure responses in two-way active avoidance (TWA) learning task**. Avoidance responses were disrupted on day-1, day-2 and day-3 in the 10 mg/kg group and were significantly different from vehicle controls (students *t*-test, *p < 0.05, **p < 0.01). Data are given as mean values ± SEM.

**Figure 4:**
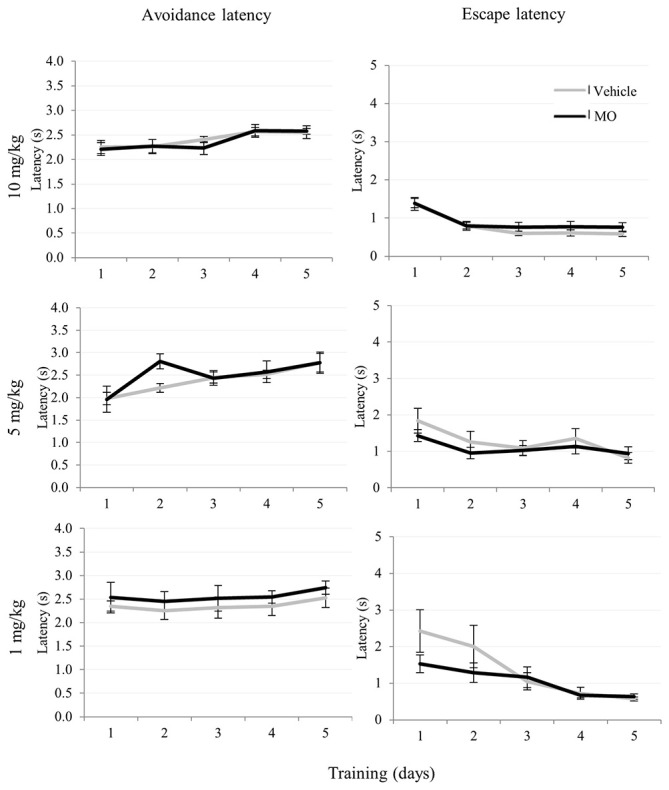
**10, 5, 1 mg/kg MO dose effects on avoidance latency and escape latency in two-way avoidance learning**. The data were shown in trend graphs with mean values ± SEM.

Comparison of drug and vehicle treated groups (Table 1) revealed significantly reduced numbers of avoidances (*F*_(1,39)_ = 12.85, *p* < 0.005) in the 10 mg/kg MO treated group compared to vehicles. There were no differences in escapes, failures, avoidance or escape latencies between vehicle treated and any of the drug treated groups (Figures 3, 4).

The day-by-day comparison for the 10 mg/kg and the vehicle treated groups indicated in Table 1 revealed significantly reduced avoidance reactions in the MO treated group on day-1 (*p* < 0.001), day-2 (*p* < 0.05), and day-3 (*p* < 0.05) compared to controls.

## Discussion

MO in clinically relevant doses impaired learning in a TWA task and enhanced long-term memory in a contextual FC task. The results of the FC learning paradigm revealed a striking difference between contextual and cued FC: while MO had an enhancing effect on contextual learning, no effect was observed for cued learning. This interesting difference is in line with a study by Shuman et al. (2009) who reported the same effect in mice even with a slightly lower dose of 0.75 mg/kg MO. The MO mediated improvement of contextual FC may indicate that the effect of the drug targets hippocampus dependent memories. In classical FC cue-shock association and context-shock association is mediated by different neuronal circuits. Contextual fear requires the hippocampus whereas the cued fear memory is generally hippocampus independent unless the task demands temporal association of CS and UCS (Anagnostaras et al., 2001; Gale et al., 2004). This hippocampus-specific effect of MO is also supported by other studies analyzing hippocampus-dependent learning paradigms. The Morris water maze is widely used to assess spatial memory in rodents which relies primarily on the hippocampal formation (Clark et al., 2005). Application of MO facilitated the water maze performance of rats by decreasing the latency and path length to reach the platform during training and increased the hippocampal long term potentiation. Furthermore, the drug was able to augment both, postsynaptic responsiveness and theta rhythm in dentate gyrus after a single application (Tsanov et al., 2010). Another study revealed that MO decreased long term memory errors in the Olton 4 × 4 maze, which infers that the drug exerts a positive effect on the visuo-spatial task that is hippocampus dependent (Burgos et al., 2010).

With respect to underlying mechanisms it appears likely that MO acts on memory acquisition of fear memory rather than on consolidation, as post-training administration of MO had no effect on test performance (Shuman et al., 2009). This interpretation is also supported by the finding that immediate fear reactions are significantly enhanced in the group treated with 10 mg/kg compared to vehicle controls after the first foot-shock pairing (i.e., during the ITI). However, there was no apparent effect after the second foot-shock pairing (i.e., during PSP). The PSP is characterized by high freezing rates in all groups of about 80% of the total time probably reaching a ceiling level during which possible differences may not be detectable.

In contrast to the learning enhancement of MO observed for FC learning paradigm, the effect of MO in the TWA learning paradigm was opposite: the avoidance responses (i.e., the parameter indicating successful learning in this task) were significantly reduced during the first 3 days of training, however, only at the highest concentration of MO. No effect was found for other parameters such as avoidance latency, number of escape reactions, escape latency or number of failures. Active avoidance learning paradigm can be fractionated into different components. It is commonly accepted that this avoidance paradigm can be subdivided into three aspects of learning process, the initial FC response (learning the association between CS and UCS shown by the freezing response, but no active behavioral response) and two main types of instrumental learning, i.e., learning of an escape reaction upon the onset of the CS, and this strategy is optimized to an active avoidance behavior. In particular, the latter requires highly complex skills which are mediated through mechanisms of working memory (Moscarello and LeDoux, 2013).

Since the cued UCS-CS associations in the FC test were unaffected by MO it is likely that the impairment in avoidance reactions in MO treated rats in the TWA is not based on impaired associative memory abilities. Moreover, the unaltered response latencies compared to the control group indicate that overall motor abilities are not affected by MO. This raises the question, which other components and/or brain structures involved in avoidance learning are affected by MO. MO inhibits the dopamine transporter and thereby increases levels of extracellular dopamine, which in turn may change the synaptic amount and assembly of dopamine receptors. *In vitro* binding assays revealed modest binding affinity of MO to the dopamine transporter with an IC_50_ value of 3.1 μM (Mignot et al., 1994). Additionally, other transmitter systems could be affected by MO, such as serotonin and noradrenalin in the prefrontal cortex (Ferraro et al., 2013). In another study, the effects of MO (128 mg/kg) on extracellular serotonin, dopamine, and noradrenaline in rat prefrontal cortex and in the medial hypothalamus area were studied. There was an initial increase of extracellular serotonin, dopamine and noradrenalin in cortex during the first 60 min following MO administration and the serotonin levels remained high until 3 h. In contrast, in the hypothalamus, only noradrenalin release was enhanced while dopamine and serotonin levels remained low (de Saint Hilaire et al., 2001).

For TWA learning *in vivo* microdialysis studies have shown that dopamine release is critical at the time point when the animal “understands” the principle of an avoidance response (Stark et al., 2000, 2004). Along the same line it has been shown that the stimulation of the ventral tegmental area improves avoidance learning (Ilango et al., 2011). Since every avoidance response acts as a reward for the animal (i.e., the animal is relieved that it does not receive the foot shock), it is tempting to speculate that chronically elevated dopamine levels mediated by MO injection hinders the pulsed release of dopamine rewarding the animal for the successful avoidance response and thereby reduces the ability to learn an active avoidance strategy. The possibility of drug state-dependent learning cannot account for the observed avoidance deficits in TWA in our study because the drug was administered during all training sessions.

Both tasks, TWA and FC require dopamine receptor activation, within the striatum, hippocampus and prefrontal cortex (Abercrombie et al., 1989; Sokolowski et al., 1994; Darvas et al., 2011; Valenti et al., 2011; Wen et al., 2015). However the relative contribution of these brain regions and their dopaminergic system may differ between tasks. Hippocampal integrity is mandatory for context, but not for cued FC, and prefrontal dopaminergic activation is indispensable for the TWA task. MO may affect dopaminergic receptor assemblies differentially in these brain regions, which may affect different memory components of task learning. It has been found that the conditioned avoidance responses were reduced when a D1 antagonist injected into the dorsolateral striatum only in the test but not the training session, whereas nucleus accumbens injections caused deficits in both sessions and systemic administration caused a combination of these effects (Wietzikoski et al., 2012). There is also evidence that synaptic localization of dopamine receptors affects avoidance behavior. The activation of post-synaptic receptors (systemically) facilitates, whereas pre-synaptic activation inhibited avoidance acquisition (Carvalho et al., 2009).

Systemic administration of Metoclopramide in rats enhanced immediate freezing in FC followed by disrupted avoidance reactions in an active avoidance test (Blackburn and Phillips, 1990). Lesions of the infralimbic prefrontal cortex prior to training increased conditioned freezing while causing a corresponding decrease in avoidance reactions (Moscarello and LeDoux, 2013). These findings indicate opposing freezing and avoidance responses induced by pharmacological intervention or brain area inactivation in active avoidance tests. However, such an effect that could potentially be caused by MO is less likely to explain the different results of the TWA and FC paradigms in our study. We found comparable means of avoidance or escape latencies in MO treated and control rats, which should be different if MO causes these effects.

Different and even opposite effects of MO upon cognitive performance in dopamine dependent active avoidance task was observed. The underlying mechanisms however are still unclear. There are at least two possible explanations: (i) chronically elevated extracellular dopamine may support task performance in the passive contextual FC but may hinder acquisition in the active avoidance task, which requires timely precise dopamine pulses for the decision making to avoid the shocks; and (ii) differential, brain site specific effects of MO and thereby relative difference in the activation of brain regions prefrontal cortex or the hippocampus upon dopamine receptor assemblies may underlie the task dependent differences in behavioral performance that we observed. Further studies to test these alternative (but not exclusive) interpretations may expand the knowledge of the underlying mechanisms of MO action in the modulation of cognitive abilities and may contribute to the understanding of the contraindicative effects of MO in anxiety related mental disorders.

## Conflict of Interest Statement

The authors declare that the research was conducted in the absence of any commercial or financial relationships that could be construed as a potential conflict of interest.

## Abbreviations

MO: Modafinil
FC: Fear conditioning
ITI: Inter-trial interval
TWA: Two-way avoidance
PSP: Post shock period
CS: Conditioned stimulus
UCS: Unconditioned stimulus.
